# Supramolecular structures: An introduction to the JBC reviews thematic series

**DOI:** 10.1016/j.jbc.2025.110905

**Published:** 2025-11-04

**Authors:** Joseph M. Jez, Wolfgang Peti

**Affiliations:** 1 Department of Biology, Washington University in St Louis, St Louis, Missouri, USA; 2 Department of Molecular Biology and Biophysics, University of Connecticut Health Center, Farmington, Connecticut, USA

**Keywords:** cryo-electron microscopy, cryo-electron tomography, protein complexes, structural biology, X-ray crystallography

The twenty years of the 21st century have been a golden age of structural biology. New instrumentation and increasing computational power are pushing the boundaries of molecular and cellular imaging. This thematic review series on "supramolecular structures" presents seven timely reviews highlighting how structural insights on large assemblies built from multiple proteins for a specific function provide new understanding of biological and chemical systems.

The molecular biology era began in the 1950s with the first views of nucleic acid (*i.e.*, DNA) and protein structure (*i.e.*, myoglobin and hemoglobin) from X-ray crystallography, as well as modeling (1). Over the next fifty years, structural biology developed the approaches and infrastructure that revealed the molecular workings of biology and chemistry across all the kingdoms of life. At the turn of the 21st century instrumental, computational, and infrastructure advances again revolutionized a the use of structural biology tools (*i.e.*, X-ray crystallography automation and newest generation synchrotron, high-field NMR and cryo-probes, cryo-EM, cryo-electron tomography) that continue to push the field forward (2, 3, 4, 5). Success is evident as more experimentally determined three-dimensional structures are now deposited annually in the Protein Data Bank than in the first 20 years of its existence (6). Importantly, these technologies, along with adventurous protein chemistry efforts and the routine ability to produce proteins in mammalian cell culture, have pushed the boundaries of structural biology beyond single macromolecules to studies that reveal the molecular details of larger and larger, and more complicated, cellular machinery.

Borrowing a term from chemistry for structures built from multiple components for a specific purpose, here, we refer to "supramolecular structures" broadly as the assemblies of macromolecules at larger scales, including those approaching what would be considered "cell biology." This review series showcases seven areas where the understanding of how individual proteins come together for different processes are expanding our structural insights on protein assemblies and their diverse biological roles.

Starting small, Wolff and Tsai (7) describe recent structural advances that provide insight on how the type II polyketide synthases (PKSs) operate as part of a larger iterative biosynthetic system. Polyketides are chemically diverse natural products found in plants and microbes. Their biosynthesis uses different classes of PKS employing distinct architectures for building intricate small molecules. For example, the type I PKS are large proteins with multiple active sites encoded on a single polypeptide (8). The active sites form an assembly line that pass a growing polyketide from active site to active site across one or more polypeptides. The simpler type II PKS use individual proteins to iteratively build extended polyketides. Although not a large complex, the individual proteins encompassing the type II PKS function together. This review underscores how molecules made by this class of PKS are important for human health and how new approaches are targeting these iterative assembly systems for their synthesis.

The review by Wang *et al.* (9) also shows how individual proteins can assemble into larger structures with dramatic consequences. They describe recent structural studies on the activation, membrane engagement, and regulation of pore-forming complexes in lytic cell death. The various types of gasdermin pore-forming proteins and the role of NINJ1, an activator of membrane rupture, are highlighted. Gasdermins create pore structures that range from 150 to 320 Å in diameter and are formed from individual proteins with 24- to 55-fold symmetry in the final assembly. These pores alter solute flow and lead to cell swelling and membrane tension changes. Upon undergoing a conformational change to gain activity, NINJ1 interacts with the gasdermin pore leading to a rupture of the cell membrane.

A different critical pore supramolecular structure is reviewed by Chien *et al.* (10), specifically the nuclear pore complex (NPC), which is the essential gatekeeper between the cytosol and the nucleus. Their review focuses on the NPC architecture and especially on recent insights on how viruses can remodel NPC architecture, change nuclear transport to their advantage, and modify the innate immune response to enhance viral replication.

The next two reviews provide examples of how conformationally flexible multidomain assemblies adapt to different cellular contexts. The first example is the leucine-rich repeat kinase 2 (LRRK2) associated with Parkinson's disease (11). LRRK2 is a ∼2500 amino acid protein with Ras-GTPase and Ser/Thr kinase domains, along with a diverse set of "repeat" domains, which modify its substrate proteins (*i.e.*, Rab GTPases). As described by Leschziner (11), the conformational versatility of LRRK2's domains adapts it to cytosolic protein interactions, association with microtubules, recruitment to cellular membranes, and activation—insights that emphasize the multimethod approach to capture key features in the formation of supramolecular assemblies in different cellular situations. This information will be valuable for developing new therapeutic approaches targeting LRRK2 and Parkinson's disease.

Similarly, adaptability in supramolecular structures is highlighted by Chen and Hurley (12). This review examines two key regulators of autophagy—unc-51-like kinase complex and class III PI3K complex I—and how their intricate regulation and activation mechanism are linked to structural changes. Both protein complexes are control switches at the start of macroautophagy, the process that maintains homeostasis in eukaryotic cells by removing unwanted and/or damaged proteins. Once again, the theme of how to build large protein assemblies from multiple polypeptides, which are conformationally flexible and adapt to different cellular roles through interactions with even larger assemblies is the theme of this review.

The last two reviews bridge protein structure to cellular function. Wiener *et al.* (13) describes recent studies on the endosome sorting complexes required for transport (ESCRT) in plants. The ESCRT proteins are critical for endocytosis and endosomal trafficking of plasma membrane proteins. Plants share conserved ESCRT systems with other eukaryotes but also have unique components for specialized processes. A combination of structural and cellular biology approaches is building new insights into the assembly of these complexes.

The final review by Biven and Wang (14) covers the largest structures (∼250 nm wide and ∼500 nm long) found in this set of reviews, centrioles and basal bodies. Centrioles are critical for spindle fiber formation during cell division. Basal bodies, which are related to centrioles, form the core of cilia and flagella assembly. In both structural assemblies, substructure differences and changes in protein composition impacts the stability, regulation, and function of these mini-organelles. The authors discuss how new imaging tools and protein identification strategies are developing detailed models for centrioles and basal bodies to better understand their dynamic composition and regulation.

Although structural studies of macromolecular assemblies have a long history, including early (and continued) efforts examining virus structure, prokaryote and eukaryote ribosomes, and assembly of replication and transcriptional machinery, the future of using all the tools of structural biology to tackle larger, technically challenging supramolecular assemblies is the next frontier for revealing the details of cellular function and regulation of various processes.

## Conflict of interest

The authors declare that they have no conflicts of interest with the contents of this article.
